# Incidentally Detected Kaposi Sarcoma of Adrenal Gland with Anaplastic Features in an HIV Negative Patient

**DOI:** 10.1155/2016/1280201

**Published:** 2016-09-26

**Authors:** Zeliha Esin Celik, Murat Celik, Erdem Sen, Hakan Cebeci, Ozlem Ata, Cagdas Yavas

**Affiliations:** ^1^Pathology Department, Faculty of Medicine, Selçuk University, Alaaddin Keykubat Campus, Selçuklu, 42000 Konya, Turkey; ^2^Pathology Department, Faculty of Medicine, Selçuk University, Turkey; ^3^Clinical Oncology Department, Faculty of Medicine, Selçuk University, Turkey; ^4^Radiology Department, Faculty of Medicine, Selçuk University, Turkey; ^5^Radiation Oncology Department, Faculty of Medicine, Selçuk University, Turkey

## Abstract

Kaposi sarcoma (KS), a vascular tumor caused by infection with human herpesvirus 8 (HHV8), is a systemic disease that can present with cutaneous lesions with or without visceral involvement. Very few cases of KS, most of which were associated with AIDS, have been reported in the adrenal gland. Anaplastic transformation of KS is a rare clinical presentation known as an aggressive disease with local recurrence and metastatic potential. We report here a 47-year-old HIV negative male presented with extra-adrenal symptoms and had an incidentally detected anaplastic adrenal KS exhibited aggressive clinical course. To the best of our knowledge, this is the first case of anaplastic primary adrenal KS without mucocutaneous involvement but subsequently developed other side adrenal metastases in an HIV negative patient.

## 1. Introduction

Kaposi sarcoma (KS) is a rare low-grade malignant vascular neoplasm caused by human herpesvirus 8 (HHV8) and often occurs in the setting of human immunodeficiency virus (HIV) infection [1]. KS is a multifocal tumor that manifests most frequently in mucocutaneous sites, typically the skin of the lower extremities, face, trunk, genitalia, and oropharyngeal mucosa [1, 2]. Visceral KS in the absence of mucocutaneous lesions is uncommon [3] and among viscera, adrenal gland involvement of KS is extremely rare with very few reports in the literature [2–6] many of which are usually associated with AIDS [1].

Anaplastic KS, which is defined histologically as a tumor with greater cytologic atypia and high mitotic rate, is accepted as a disease with a more locally aggressive behaviour and metastatic potential, but its exact incidence is indeterminate in the literature [1, 7].

Herein, we report a case of anaplastic primary adrenal KS without mucocutaneous involvement which is incidentally diagnosed in an HIV negative patient and exhibited aggressive clinical course with a metastatic tumor detected at the other adrenal gland seven months after the initial diagnosis.

## 2. Case Report

**Figure 1:**
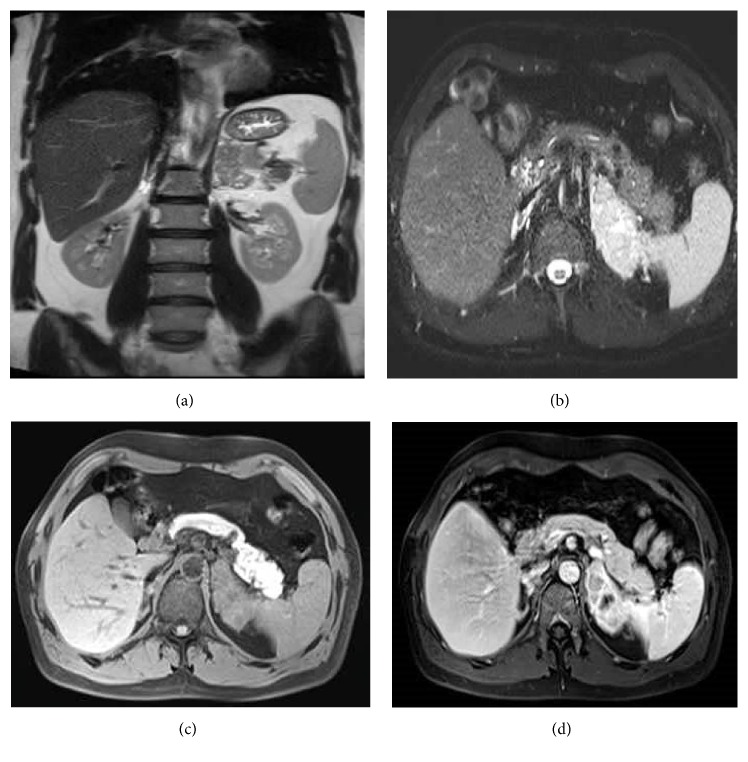
(a) Coronal T2-weighted and (b) axial fat saturated T2-weighted images show slightly hyperintense left adrenal gland lesion by comparison to spleen, with cystic degenerative components. (c) Axial fat saturated nonenhanced T1-weighted and (d) axial fat saturated enhanced T1-weighted enhanced images reveal diffuse and heterogeneous enhancement of lesion.

**Figure 2:**
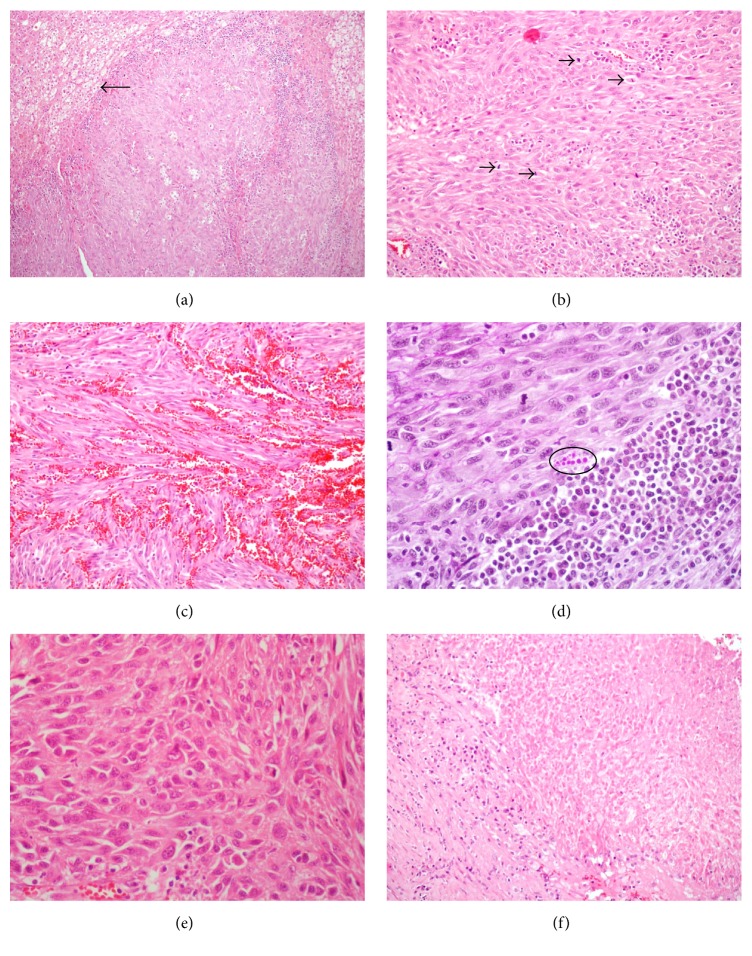
(a) Normal adrenal cortex parenchyma (arrow) invaded by tumor (hematoxylin-eosin ×40). (b) Tumor composed of short fascicles and storiform patterns of spindle or epithelioid cells with oval-round nuclei and eosinophilic fibrillary cytoplasm. Increased mitosis is also seen (arrows) (hematoxylin-eosin ×100). (c) Slit-like spaces and extravasated red blood cells within the tumor cells (hematoxylin-eosin ×200). (d) PAS positive intracytoplasmic hyaline globules in some tumor cells (circle) (×400). (e) Epithelioid tumor cells with nuclear atypia and pleomorphism (hematoxylin-eosin ×400). (f) Foci of necrosis are present in some areas (hematoxylin-eosin ×200).

**Figure 3:**
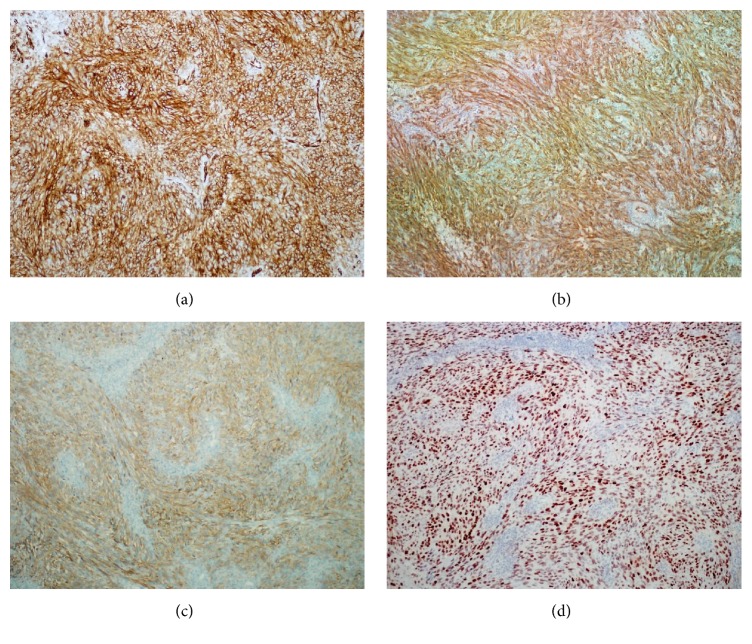
Diffuse positive staining of tumor cells immunohistochemically with (a) CD31, (b) CD34, (c) D2-40, and (d) HHV8 (×100).

A 47-year-old male applied to a pulmonologist with complaint of cough. Thorax computed tomography was performed and 8 × 6 × 3 cm mass was incidentally detected at left adrenal gland on radiological sections. Then, the patient was evaluated in terms of hormonal activity of the mass by endocrinologist with suspicion of pheochromocytoma, Cushing syndrome, or hyperaldosteronism but the results were negative for each. Subsequently, a dynamic magnetic resonance imaging of left adrenal gland was performed and T1-weighted enhanced images revealed diffuse and heterogeneous enhancement of lesion (Figure 1). These results led to a suspicion of malignancy and a fluorodeoxyglucose positron emission tomography computed tomography (FDG PET/CT) was performed for malignancy screening. FDG PET/CT examination revealed an increased 18F-FDG uptake in the left adrenal gland with a maximum standardized uptake value (SUVmax) of 19.60. Increased 18F-FDG uptake was not detected anywhere else. In the light of these findings, the patient was operated on with a preoperative diagnosis of adrenal carcinoma. A left laparoscopic adrenalectomy was performed. Left adrenal mass was completely excised without any lymph node excision. On gross examination, a 55 g weighted, 8.0 × 6.0 × 3.0 cm sized gray-white solid mass with irregularities on outer surface was seen. On cut surface, yellow areas resembling normal adrenal tissue invaded by numerous nodules with 0.3–1.5 cm diameter were detected. Multiple samples were taken from the mass for microscopic evaluation. On microscopic examination, a tumor invading adrenal cortex parenchyma (Figure 2(a)) with focally positive margins and extracapsular infiltration composed of short fascicles and storiform patterns of spindle or epithelioid cells with oval-round nuclei and eosinophilic fibrillary cytoplasm was seen (Figure 2(b)). Within the tumor cells, slit-like spaces and extravasated red blood cells were present (Figure 2(c)). The tumor was divided into nodules by fibrous septa and lymphoplasmacytic inflammatory cells. PAS positive intracytoplasmic hyaline globules were detected in some tumor cells (Figure 2(d)). In some areas, tumor cells exhibited severe nuclear atypia and pleomorphism (Figure 2(e)). Mitotic count was 18/10 HPF. Additionally, hyalinisation and foci of necrosis were present (Figure 2(f)). Immunohistochemically, tumor cells demonstrated diffuse positive staining with vimentin, CD31, CD34, and D2-40 favoring their vascular origin (Figures 3(a), 3(b), and 3(c)). Diffuse and strong nuclear HHV8 positivity was the diagnostic clue (Figure 3(d)). Some tumor cells also showed focal and weak positivity with S100 and smooth muscle actin. Negative staining was obtained with Pan-cytokeratin, desmin, Melan-A, CD99, alpha inhibin, bcl-2, synaptophysin, chromogranin, and calretinin. Ki 67 proliferation index was 95%. Histopathological and immunohistochemical features were consistent with “Kaposi sarcoma-anaplastic variant.” Approximately one month later, the patient applied to oncology clinic. Blood tests performed with microparticle enzyme immunoassay (MEIA) method prior to treatment revealed that the patient's HIV was seronegative. No accompanying mucocutaneous lesions including skin and oral or nasopharyngeal mucosa were detected on physical examination. Upper and lower GI endoscopy performed 6 months earlier due to GI symptoms were negative for any gastric or bowel mass. Self- or family history did not include any additional features. During his evaluation, abdomen computed tomography revealed a 2.8 × 1.4 cm mass, which invaded the spleen and left renal artery resulting in cortical infarct of left kidney, at the operation site consistent with recurrence of KS. The patient had underwent surgery again. Pathological examination revealed splenic invasion and metastasis of KS in multiple lymph nodes located at perisplenic and left adrenal region. Postoperatively, patient had adjuvant radiotherapy with weekly cisplatin (50 mg total/week). Two months after radiotherapy, all imaging studies were negative for recurrence and metastasis. Seven months after initial diagnosis, on abdominal MRI, a 2.5 × 2 cm tumor radiologically consistent with Kaposi sarcoma was detected at the other adrenal gland. FDG PET/CT revealed an increased 18F-FDG uptake in the right adrenal gland with a SUVmax of 15.84 suggestive of KS metastasis. The patient underwent surgery again. The pathological diagnosis of the excised tumor was Kaposi sarcoma of the right adrenal gland. Chemoradiotherapy was planned again.

## 3. Discussion

KS, an unusual tumor described more than 100 years ago, presents in various clinical forms including classic (sporadic), endemic (African), iatrogenic (transplantation-associated), and epidemic (AIDS-related) KS [1, 8, 9]. Despite these different clinical subtypes, epidemiologic data strongly pointed to an infectious etiology for all. The agent, subsequently classified as a gamma 2 herpesvirus, is known as Kaposi sarcoma-associated herpesvirus (KSHV) or human herpesvirus 8 (HHV8) [9].

KS has been first described in five cases principally affecting the skin of the lower extremities of elderly men by Kaposi in 1872 [9]. KS represents mainly as mucocutaneous lesions but may also involve lymph nodes [2] and less frequently other anatomic locations such as the musculoskeletal system, urinary system, respiratory and gastrointestinal tracts, nervous system, larynx, eye, major salivary glands, heart, breast, and endocrine glands including the adrenal gland and thyroid [1, 2, 10, 11]. The development of KS within wounds and blood clots is also presented. Chen et al. [11] presented a non-AIDS-related primary intraosseous KS. KS in these atypical sites may prove to be difficult to diagnose, resulting in patient mismanagement [2].

In the current literature, there have been few numbers of case reports of adrenal KS most of which were described in patients with AIDS [1–3]. In postmortem studies, adrenal KS have been reported in about 20% of patients with classic (sporadic), African (endemic), and AIDS-related (epidemic) KS for each clinical subgroup [2, 12]. The predilection of KS involvement in adrenal gland seems to be adrenal cortex rather than medulla [2, 13]. Rotterdam and Dembitzer [4] examined the adrenal gland of 66 AIDS patients autopsied and reported only one patient with KS. In another autopsy study of 100 AIDS patients, Bricaire et al. [6] documented only 3 cases of adrenal KS. Lazure et al. [3] described a case of bilateral adrenal KS in an HIV seronegative black patient as a very extreme presentation. The present case demonstrating bilateral Kaposi sarcoma of the adrenal gland is another example for this extremely rare appearance.

Regardless of the clinical subgroup, the histological appearances of KS are similar [1, 7, 8]. Three distinctive stages, which can overlap, are described according to the evolution of a particular lesion: patch, plaque, and nodule [8]. The early patch stage is characterized by proliferation of small slit-like or ectatic vascular spaces, which dissect between dermal collagen, mostly in the upper dermis [1, 7]. These vascular channels are lined by flattened endothelial cells, with a mild inflammatory infiltrate composed mostly of lymphocytes and plasma cells [1]. As early changes in the patch stage can be very subtle, confusion with an inflammatory dermatosis is possible [8]. The more advanced plaque stage is characterized by a proliferation of spindle shaped cells arranged as short fascicles and a diffuse proliferation of vessels [7]. The nodular stage of KS is even more cellular and characterized by well defined nodules of vascular spaces which replace the dermis and spindle shaped cells which usually show minimal cytologic atypia and frequent mitotic figures. Slit-like spaces containing red blood cells and intracellular or extracellular periodic acid Schiff (PAS) positive eosinophilic hyaline globules are easily identified at this stage of the disease [1, 7, 8]. Although present in all types of KS, these globules, measuring from 0.4 to 10 mm and probably representing degenerate red blood cells, are more frequent in AIDS-related KS [8]. In the present case, both spindle and epithelioid cell morphology were detected and PAS positive hyaline globules were easily found in some tumor cells.

An anaplastic variant of KS is mainly reported in African cases some years ago and only small series on anaplastic transformation of classic KS has been encountered [8, 9]. Anaplastic variant of KS has been first described by Cox and Helwig as a tumor exhibiting increased mitosis and severe cellular pleomorphism [1, 9]. Skin is the most common involved site by anaplastic KS, followed by lymph nodes, liver, and spleen less frequently [1]. Huwait et al. [1] reported a case of adrenal gland located KS with anaplastic morphology in a patient with AIDS. In our case, the diagnosis was anaplastic KS as some tumor cells showed significant pleomorphism and increased mitosis. In our patient, the disease exhibited aggressive clinical behaviour, favoring its anaplastic nature, with development of splenic invasion, lymph node metastasis, and left renal artery involvement only one month after the diagnosis and detection of a metastatic tumor at the other adrenal gland seven months after the initial diagnosis.

Demonstration of HHV8 in lesions of KS by in situ hybridisation (ISH) and recently immunohistochemical methods using a monoclonal antibody against the latency associated nuclear antigen-1 (LATA-1) of HHV8, which can be used in paraffin-embedded tissues, is an invaluable tool in the histologic diagnosis of KS, as this marker is consistently positive in all clinical variants of the disease. Furthermore, it is helpful in differentiating KS from other vascular proliferations which are only exceptionally positive for HHV8 [1, 7, 8].

The differential diagnosis of KS includes many benign and malignant entities depending on its location. KS located at the skin must be distinguished from hemangiomas, lymphangiomas, and their variants, as well as angiosarcoma [7–9]. In our patient, the principal differential diagnosis included adrenal cortical adenoma, carcinoma, and pheochromocytoma. Immunohistochemically, negative staining of tumor cells with alpha inhibin, synaptophysin, and chromogranin was helpful to exclude these tumors. Spindle and epithelioid cell morphology with some degree of pleomorphism and increased mitotic count raised a suspicion of sarcoma or melanoma, but immunohistochemistry including desmin, CD 99, calretinin, and Melan-A was not supportive. The presence of slit-like spaces and extravasated red blood cells was suggestive of a vascular tumor. Diffuse positive staining of tumor cells with CD31, CD34, and D2-40 favored their vascular origin. Diffuse and strong nuclear HHV8 positivity was the diagnostic feature of KS in our case.

In conclusion, we report a 47-year-old man with a left adrenal anaplastic KS demonstrating an aggressive clinical course with a metastatic tumor detected at the other adrenal gland. To the best of our knowledge, this is the first case of anaplastic primary adrenal KS without mucocutaneous involvement in an HIV negative patient. Although extremely rare, KS must be kept in mind in differential diagnosis of adrenal lesions exhibiting KS-like features histologically. Immunohistochemical stains including HHV8 may be helpful in differential diagnosis and establishing accurate diagnosis.
